# The Sufferings of Christ

**Published:** 1887-08-27

**Authors:** 


### WORDS OF CONSOLATION.

[Communications for this column should be addressed, "Chaplain," care of the Editor of THE HOSPITAL, who will gratefully welcome any suggestions or inquiries from matrons, nurses, and the staff generally.]

### XLVII.—THE SUFFERINGS OF CHRIST.

(*For Reading to the Sick.*)

ST. PAUL speaks about "Knowing the fellowship of Christ's sufferings" (PHIL. iii, 10). This must mean having some fellowship with Him in His Agony, Passion, and Death, a fellowship which implies some deep sympathy between the member and the Head. This was what made St. Paul such a changed man. "Saul, Saul, why persecutest thou Me?" (ACTS ix, 4). God revealed to him at his conversion that it was Jesus whom he had wounded by his sins. It was evident that even after our Lord had returned to Heaven the wounds in His tender heart could be re-opened. He can be crucified afresh. We make a great mistake if we regard our Lord's Passion just as if it were all past and done in such a way that He can never feel for sin any more. Certainly, He can never lie prostrate, nor sweat drops of blood in that garden again. He will never be scourged, nor tortured in the body, nor be nailed literally to the Cross again. But remember, the very same Soul which endured so much for you, is still as sensitive as ever, still as full of feeling, still in some mysterious way is capable of being grieved on account of sin. We ought to be sorry indeed for all that He endured upon the Cross; for if we had not sinned He need not have died. But it is yet more practical to be certain that we cannot sin now without wounding Him again. All wilful sin, either in thought, word, or deed, is like a crucifying afresh of the Son of God. Every time He has called us to Himself, and we have known that He has been standing knocking at the door, and yet turned a deaf ear to His entreaty, we have caused Him pain. Neglect of religion as well, without any deliberate wickedness, is quite enough to grieve One who loves so tenderly. You know well the uncomfortable feeling when you have become conscious of having caused a dear friend unnecessary pain. If only through some mistake or misunderstanding you have afflicted the soul of some one very dear to you, you have had no rest until the matter was set right. You have thought, "I cannot bear giving pain to one who loves me so dearly." That husband is hard indeed who is continually annoying his wife. That mother is unnatural who can see her innocent child suffer and care not for it. That son has a heart of stone who can hear that his obstinacy or prodigality has caused his parents sorrow upon sorrow, and yet not be moved. Ah, yes! but remember, there is One Heart tenderer than any of these, One that has bled and been broken, been sorrowful even unto death for you. There is One who may be grieving on your account even now. Suppose you could hear Him speaking at this moment, is it possible that His words to you would be such as the Church uses year after year from the Lamentations of Jeremiah, in order to bring many of her more forgetful children to a due sense of their Saviour's Passion?—"Is it nothing to you, all ye that pass by? Behold, and see if there be any sorrow like unto my sorrow, which is done unto me, wherewith the Lord hath afflicted me in the day of his fierce anger" (LAM. i, 12).

(*To be continued.*)

